# Microscopic polyangiitis in pediatric systemic lupus erythematosus: a unique presentation of pulmonary-renal syndrome and case report of an overlap syndrome

**DOI:** 10.1007/s13730-024-00949-0

**Published:** 2024-12-11

**Authors:** Chen-xing Zhang, Lei Yin, You-ying Mao, Zheng-yu Zhou, Wei Zhou

**Affiliations:** Department of Nephrology, Shanghai Children’s Medical Center, Shanghai Jiao Tong University School of Medicine, 1678 Dongfang Road, Shanghai, China

**Keywords:** Systemic lupus erythematosus (SLE), Antineutrophil cytoplasmic autoantibodies associated vasculitis (AAV), Overlap syndromes

## Abstract

Secondary vasculitis is encountered in about one-third of all cases of systemic lupus erythematosus (SLE). Skin is most commonly involved in lupus-related small vasculitis. Although antineutrophil cytoplasmic autoantibodies (ANCA) associated vasculitis (AAV) is relatively uncommon, it can be the most dangerous manifestation associated with high mortality. SLE and AAV are separate diseases with different pathophysiologies and an overlap syndrome has only been reported a few times in previous literature. We present a unique case of a pediatric patient of pulmonary-renal syndrome, presenting with pulmonary alveolar hemorrhage and rapidly progressive glomerulonephritis. Serological and biopsy findings were suggestive of SLE and AAV occurring, simultaneously. Renal biopsy demonstrated necrotizing and crescentic glomerulonephritis, superimposed on diffuse segmental proliferative lupus glomerulonephritis class IV. The presentations of autoimmune diseases and vasculitis can be multi-systemic. Considering overlap syndromes, especially in patients with underlying connective tissue disease or systemic vasculitis, is vital for prompt therapy and prevention of morbidity in this population.

## Introduction

Systemic lupus erythematosus (SLE) and ANCA-associated vasculitis (AAV) are separate diseases with different pathophysiologies. While both affect the kidneys and other systemic involvements, SLE can lead to an immune complex glomerulonephritis, while AAV is characterized by pauci-immune necrotizing and crescentic glomerulonephritis [1, 2]. Pulmonary-renal syndrome, for instance, diffuse alveolar hemorrhage (DAH) and rapidly progressive glomerulonephritis can occur in both of the diseases [3, 4]. The occurrence of both SLE and AAV simultaneously has only been depicted few times in previous literature, especially in the pediatric population. Serological tests are extremely helpful when making a diagnosis, but ultimately it must be based on the results of renal biopsy. Treatment with corticosteroids, immunosuppressive agents, and, in severe cases, plasmapheresis has considerably improved the prognosis [5, 6]. Here, we have reported the case of a 14-year-old girl with an overlap syndrome of SLE and AAV and reviewed her kidney biopsy finding, the differential diagnosis, diagnosis as well as the management of the disease.

## Case report

A 14-year-old girl was admitted to our hospital because of arthralgias, rash, and fever. The patient had been well until 1 month before admission, when arthralgias affecting knees and ankles, as well as rash in lower extremities developed. One day before admission, she had a fever with peak temperature of 38.6 °C. The patient appeared ill, with pale face and lip. She had known allergy of penicillin, with no history of recent medication, travel, exposure to sick persons, blood transfusions, major illness or previous surgery. She had no previous personal or family history of autoimmune disease, except that her mother suffered from hyperthyroidism.

On physical examination, the temperature was 38.7 °C, the blood pressure 108/60 mmHg, the pulse 90 beats per minute, the respiratory rate 26 breaths per minute, and the oxygen saturation 100% while she was breathing ambient air. The height was 158 cm, the weight 45 kg, and the body-mass index (the weight in kilograms divided by the square of the height in meters) 18.02. There was dependent edema and dispersive purpura like rash over both of the lower extremities. The auscultation of the chest revealed wet rales in the right lung. The review of other systems was negative. Initial laboratory examinations revealed anemia with hemoglobin of 8.2 g/dl, kidney insufficiency with a creatinine of 120 μmol/l, reduced albumin level of 28.3 g/l as well as elevated inflammatory markers. Urinalysis revealed proteinuria and hematuria.

**Fig. 1:**
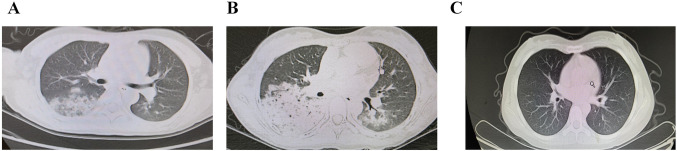
High-resolution computed tomography (HRCT) of bilateral alveolar infiltrates is suggestive of diffuse alveolar hemorrhage. HRCT of the chest showed multiple patches with increases in density and peripheral opacities in the 2nd hospital day (**A**). There was massive infiltrates in right lobes, accompanied by pleural effusions in the fourth hospital day, which demonstrated an obvious radiological progression (**B**). There was neither inflammatory exudation nor pleural effusion, as demonstrated by chest CT re-examination in the 4th month of the follow up (**C**)

A cough developed during the next day. The cough was intermittently productive of thick white sputum that occasionally contained a small amount of blood. The patient also reported fatigue, episodes of shortness of breath and transient chest pain, without headaches, abdomen pain, visual loss, dry eyes or dry mouth. Thus high-resolution computed tomography (HRCT) of the chest was performed. CT of the chest showed multiple patches with increases in density and peripheral opacities (Fig. 1A). Axillary lymphadenopathy was also identified. Ceftazidime and azithromycin was administered intravenously. Supplemental oxygen was also administered through a nasal cannula at a rate of two liters per minute.

Subsequent blood work revealed low C3 (0.87 g/l), ANA (1:100), p-ANCA, anti-myeloperoxidase (MPO), anti-SSA auto-antibody as well as positive Coombs test. Moderate schizocyte were identified in peripheral blood smear test. Anti-glomerular basement membrane (GBM) antibody, and antibody to proteinase 3 (PR-3) were negative. HIV, HBV, and HCV were also tested to be negative. The comprehensive laboratory test results are shown in Table 1. The ultrasonography of the abdomen revealed thickening of liver parenchyma. The ultrasonography of the urinary system revealed diffuse alterations in bilateral renal cortex. An electrocardiogram (ECG) showed sinus arrhythmia and nonspecific T-wave abnormalities. Cardiac ultrasonography revealed normal global cardiac function and right-ventricular size, no evidence of a pericardial effusion.

**Table 1:** Laboratory test results during the disease course

Variable	On admission	Before plasma exchange	Before discharge
White cell count (per mm^3^)	7250	13,150	10,550
Differential count (%)			
Neutrophils	75.5	85.5	76.2
Lymphocytes	17.5	11.2	17.4
Eosinophils	0.4	0	0.4
Platelet count (per mm^3^)	193,000	253,000	277,000
Hemoglobin (g/dl)	8.2	6.4	11.8
d-Dimer (mg/l)	1.81	1.55	0.44
Activated partial-thromboplastin time (s)	34.5	28.9	24
Prothrombin time (s)	10.9	10.7	9.6
International normalized ratio for prothrombin time	1.01	0.99	0.89
Fibrinogen (g/l)	4.3	2.98	2.01
Carbon dioxide combining power (mmol/l)	18	18	20
Creatinine (μmol/l)	120	128	76
Albumin (g/l)	28.3	22.2	30.3
Ferritin (ng/ml)	–	125.3	125
C reactive protein (mg/l)	16	3	1
Erythrocyte sedimentation rate (mm/h)	92	–	7
Antinuclear antibody	1:100	–	1:100
Anti-RNP antibody (U/ml)	Negative	–	Negative
Anti-Smith antibody (U/ml)	Negative	–	Negative
Anti-SS-A antibody (U/ml)	Positive	–	Positive
Anti–SS-B antibody (U/ml)	Negative	–	Negative
Anti-double-stranded DNA	Negative	–	Negative
Anticardiolipin antibody (U/ml)	Negative	–	–
p-ANCA	Positive	–	Positive
c-ANCA	Negative	–	Negative
Anti-MPO	Positive	–	Positive
Anti-PR3	Negative	–	Negative
Complement (g/l)			
C3	0.87	–	1.06
C4	0.15	–	0.19
*Urine*			
Screening-dipstick test			
Protein	3+	3+	3+
White cells	Negative	Negative	Negative
Red cells (per high-power field)	Full field of vision	Full field of vision	25–28
24 h urine protein (mg/24 h)	3540	–	4338
Blood culture	Negative	–	–
Epstein-Barr DNA	Negative	–	–
Cytomegalovirus DNA	Negative	–	–
Coombs test			
Direct Coombs test	Positive	–	Negative
Indirect Coombs test	Negative	–	Negative

The patient met 4 of the 11 lupus criteria based on the 1997 American College of Rheumatology criteria: hematological disorder (hemolytic anemia), kidney disorder (proteinuria of 3.54 g in 24 h), arthritis and positive ANA. She also met 5 of the 17 lupus criteria according to the 2012 Systemic Lupus International Collaborating Clinics (SLICC) classification criteria: arthritis, hemolytic anemia, kidney disorder (proteinuria of > 500 mg per 24 h), positive ANA, and low complement (low C3), which includes three clinical and two immunological items. Considering involvement of renal and pulmonary system is due to the occurrence of primary disease of lupus, methylprednisolone (500 mg, 1 g and 1 g daily for 3 days) was administered.

Fever dropped to normal and was stable in the third hospital day. On the fourth hospital day, the patient appeared to be fatigued, the oxygen saturation fell to 93% with accelerated breathing (30 breaths per minute). Because of persistent respiratory symptoms, repeated chest CT scanning was performed. There was massive infiltrates in right lobes, accompanied by pleural effusions (Fig. 1B). Oxygen (5 l per minute) was administered by face mask and she was transferred to the pediatric intensive care unit (PICU) immediately due to the concurrent deteriorating kidney function, implied by the increasing serum creatinine levels. On examination, the patient appeared chronically ill. The temperature was 36.8 °C, the pulse 70 beats per minute, the blood pressure 133/88 mmHg, the respiratory rate 19 breaths per minute, and the oxygen saturation 96% while she was receiving supplemental oxygen through a face mask at a rate of 5 l per minute. Auscultation of the lungs revealed scattered bilateral crackles. The treatment with ceftazidime and azithromycin was discontinued and treatment with meropenem, pulse cyclophosphamide, and administration of methylprednisolone was initiated. In addition, a femoral vein catheter was placed, plasmapheresis was begun and fentanyl was administered. After two sessions of plasmapheresis, improvement in kidney function and resolution of hemoptysis was observed. The patient underwent 4 units of packed red blood cell transfusion in the previous two days during the PICU stay. She had a total of seven sessions of plasmapheresis.

After seven sessions of plasmapheresis, there was an improvement in kidney function. The patient’s condition is relatively stable, without requirements for oxygen. Then renal biopsy was performed. The examination of formalin-fixed tissue by means of light microscopy showed proliferative-appearing glomeruli with focal formation of fibrous and fibrocellular crescents. The GBM was not thickened. Some glomeruli were globally or segmentally sclerosed, indicating disease chronicity. The renal tubules and the interstitium were also abnormal, with mild focal tubular atrophy and mild interstitial focal fibrous hyperplasia and an inflammatory infiltrate composed primarily of monocytes and lymphocytes. Immunofluorescence staining of frozen tissue showed staining with IgG (+~++), IgM (+~++), IgA (+~++), C3 (++), C1q (+~++), Fn (++).

The classification of lupus nephritis is based on the glomeruli alone. In this case, more than half the glomeruli were diseased (indicating diffuse glomerulonephritis) and there was segmental involvement, so the disease was classified as a diffuse segmental proliferative lupus glomerulonephritis, with crescent formation and highly suspected of focus of necrosis, referred to as class IV-S (A/C) (ISN/RPS2003). The activity score is 9 and the chronicity index is 5.

Since ANCA-associated vasculitis is the most common cause of the pulmonary-renal syndrome, the diagnosis must be considered in this patient. Of patients with granulomatosis with polyangiitis (GPA) and microscopic polyangiitis (MPA), 85–90% have ANCA, namely anti-myeloperoxidase (MPO) or PR3 antibodies [7, 8]. The systemic features, such as fever, arthralgias and skin lesions (palpable purpura) are also common and were present in this patient. The pulmonary lesion in ANCA-associated vasculitis is DAH or focal inflammatory vasculitis. In GPA, there may also be lesions in the upper airways (tracheitis, sinusitis, and otitis), necrotic pulmonary lesions, and hilar lymphadenopathy [5]. The absence of asthma, sinus disease, and lung nodules rules out GPA or eosinophilic granulomatosis with polyangiitis (EGPA). Histopathological confirmation of the diagnosis of AAV is highly desirable and tissue biopsy can be important for the diagnosis and staging of AAV. In this patient, the renal biopsy demonstrated crescent formation and focus of necrosis, which was in accordance with the pathogenic features of MPA. In addition, the case met the 2017 ACR/EULAR classification criteria of MPA.

SLE with both renal and pulmonary involvement should be given serious consideration in this patient, even though DAH occurs in only 0.5–5.4% of patients with SLE [9–12]. DAH is usually associated with lupus nephritis and antiphospholipid autoantibodies in SLE patients [13–15]. The exact cause of DAH pathology remains unknown, the lung lesions can be bland alveolar hemorrhage or a localized immune complex-induced pulmonary capillaritis, contributing to damage to basement membranes and leakage of erythrocytes into the alveolar space [16]. This patient had evidence of both hemorrhage and inflammation. The patient met the 1997 American College of Rheumatology criteria as well as the 2012 SLICC classification criteria for lupus, as mentioned above [17, 18]. We concluded that alveolar hemorrhage and nephritis is due to SLE or AAV. Therefore, the diagnosis of the patient is SLE with a diffuse segmental proliferative lupus nephritis, with crescent formation and highly suspected of focus of necrosis, referred to as class IV-S (A/C) (ISN/RPS2003) as well as MPA.

The fever, cough, hemoptysis, and new pleural effusion on repeated HRCT examination may reflect an underlying respiratory infection which include those caused by bacteria such as bacterial endocarditis, legionnaires’ disease, mycobacterium tuberculosis infection, as well as mycoplasma pneumoniae (MP), and fungal infection. The negative results of blood culture and relevant etiology (negative TSPOT and MP mRNA, normal blood levels of 1,3-β-d-glucan) excluded these possibilities. Other viral infections were ruled out by the absence of typical clinical features and negative serologic tests including viruses such as HIV, EBV, CMV, hepatitis B, and C. The differential diagnosis is depicted in Table 2. Yet the most likely diagnosis was either SLE or microscopic polyarteritis (MPA).

**Table 2:** Serological and pathological features of 28 cases of AAV/SLE overlap syndrome

	Age	Sex	Hematuria	Proteinuria (mg/d)	Serum creatinine (mg/dL)	ANA	Anti- dsDNA	Low C3/C4	ANCA	Renal biopsy immunofluorescence
[36]	35	F	Yes	ND (2+)	2.3	1:320	18.2	Yes/no	MPO(+)	IgM+ C3c+
[37]	59	F	Yes	2081	2.4	1:80	54.9	Yes/yes	MPO(+)	C3+
[38]	44	F	Yes	ND (2+)	2.9	1:40	(−)	Yes/yes	MPO(+)	IgG 2+, IgA 1+, IgM 1+, C3 2+
[39]	77	M	Yes	887	3.5	(+)	(+)	Yes/no	MPO(+)	No immune deposits
[40]	40	F	Yes	ND(3+)	8.7	1:640	ND	ND	MPO(+)	Pauci-immune
[41]	40	F	ND	ND	8.7	1:640	(−)	ND	MPO(+)	ND
[42]	48	F	Yes	600	ND	1:40	(−)	No/no	PR3(+)	IgG+ C3+
Case 1 of [34]	22	F	Yes	1500	2.7	1:1286	(−)	ND	MPO(+)	IgG 3+ IgM 2+ IgA+ C3 3+ C1q 2+
Case 2 of [34]	37	F	Yes	5900	10	1:80	(+)	ND	MPO(+)	IgG 3+ C3 3+ C1q 1+
Case 3 of [34]	62	F	Yes	2000	4.7	1:848	(+)	ND	(−)	IgG+ IgM+ C3 2+
Case 4 of [34]	80	F	Yes	4000	4.5	1:2560	(−)	ND	(−)	IgG+ IgM+
Case 5 of [34]	19	M	Yes	4000	9.6	1:160	(+)	ND	(−)	IgG 3+ IgA+ C3 2+ C1q 2+
Case 6 of [34]	50	F	Yes	3200	4.4	1:160	(−)	ND	MPO	IgG3+ IgA+ C3 2+ C1q 2+
Case 7 of [34]	55	M	Yes	3000	21	1:160	(+)	ND	(−)	IgG 2+ C3 2+
Case 8 of [34]	37	F	Yes	6400	1.1	1:40	(−)	ND	MPO	IgG 3+ C3 2+
Case 9 of [34]	44	F	Yes	1700	8.8	1:320	(+)	ND	(−)	IgG 2+ IgM 1+ IgA 1+ C3 2+
Case 10 of [34]	78	F	Yes	3000	4.5	1:640	(−)	ND	MPO(+), PR3(+)	IgG 2+ C3 2+
Case 1 of [33]	23	F	Yes	800	1.02	1:800	23	No/yes	(−)	C3+
Case 2 of [33]	43	F	Yes	3200	7.11	1:1280	(−)	No/no	MPO(+)	IgG+ IgM+ C3+
Case 3 of [33]	57	F	Yes	3600	5.63	1:1280	(−)	No/no	MPO(+)	Pauci-immune
Case 4 of [33]	29	F	Yes	300	1.01	1:640	20	No/no	MPO(+)	IgG 3+ IgM 2+ C1q 2+
Case 5 of [33]	41	F	Yes	4000	1.53	1:2560	50	Yes/yes	MPO(+)	IgG 2+ IgA 2+ C3 3+ C1q 2+
Case 6 of [33]	53	F	Yes	6000	1.62	1:600	(−)	Yes/yes	MPO(+)	(−)
Case 7 of [33]	27	F	Yes	800	3.93	1:200	22	No/yes	MPO(+)	IgG+ C3+
Case 8 of [33]	74	F	Yes	1000	3.31	1:5240	50	No/no	MPO(+)	IgG 2+ C3 2+
Case 1 of [35]	74	F	Yes	600	4.1	1:5120	(+)	No/yes	MPO(+)	IgG+ IgM+ C3+
Case 2 of [35]	35	F	Yes	> 6000	3.4	1:620	(+)	No/no	MPO(+)	IgG+
Case 3 of [35]	21	F	Yes	2500	3.8	1:2560	(+)	No/no	MPO(+)	IgG+ IgM+ C3+ C1q+

Since the presence of active lupus nephritis (LN) and MPA was confirmed, the patient was then given the second round of methylprednisolone pulse therapy (500 mg daily) for 3 days as well as cyclophosphamide intravenously (400 mg daily for 2 days) every 4 weeks. The patient did follow up with the nephrology and rheumatology department, and therefore, received the additional planned cyclophosphamide infusions while taking oral hydroxychloroquine and prednisone. She has completed the sixth round of cyclophosphamide infusions and was on maintenance therapy with mycophenolate mofetil (MMF, 0.75 g + 0.5 g, bid) up to now. She had a favorable renal response, with drop of proteinuria from initial 6781 mg/24 h to present 1089 mg/24 h. Her kidney function also continued to improve, and 4 months after discharge, the creatinine was 6.67 mg/L (59 μmol/l). Her serum albumin (36 g/l) and hemoglobulin (115 g/l) both recovered and has remained stable. In regard to her pulmonary status, there was neither inflammatory exudation nor pleural effusion, as demonstrated by chest CT re-examination (Fig. 1C). She adhered well to the medications and the prednisone dose was tapered gradually from 25 mg/bid to 15 mg/qd.

## Discussion

The involvement of mucocutaneous, musculoskeletal, respiratory, and urinary system suggested a systemic illness affecting multiple systems. The differential diagnosis includes systemic multisystem illness, such as infectious diseases, malignant tumors, immune-mediated disorders, and other disorders. Yet the most likely diagnosis was either SLE or MPA.

SLE is a typical autoimmune disease characterized by chronic inflammation, pathogenic auto-antibodies, and multi-systemic involvement, with a wide range of clinical presentations including rashes, arthralgias, pleuritis, or kidney disorders [2]. Our patient tested positive for ANA, p-ANCA, anti-SSA and had low C3 with elevated inflammatory markers and biopsy proven Class IV lupus nephritis. Our patient met both lupus criteria based on the 1997 American College of Rheumatology criteria and the 2012 SLICC classification criteria. Thus SLE was primarily considered when we initially reviewed the case.

While there are no specific classification criteria for pediatric MPA, the Chapel Hill nomenclature provides a useful definition for clinical studies in children [19]. The additional clinical features of pulmonary-renal syndrome and laboratory findings in this case also pointed strongly toward the underlying MPA. The diffuse changes in bilateral renal cortex implied that the disease may be chronic, as evidenced by ultrasound of urinary system. Since abnormal kidney echotexture and progressive kidney insufficiency are ominous signs, a renal-biopsy specimen would be crucial in the determination of the diagnosis, the prognosis, and the best course of therapy.

The renal biopsy revealed immune-complex deposits consistent with WHO class IV diffuse segmental proliferative lupus glomerulonephritis [20]. Seventy percent of glomeruli (7/10) was circumferential fibrous crescents and fibrocellular crescents in our patient, the proportion of which is much higher than that observed in the general SLE population. Nevertheless, necrotizing and crescentic glomerulonephritis (including cellular and fibrocellular and fibrous crescents), one of the features and usually present in renal biopsy of AAV patients, was also observed in our patient. Based on the Chapel Hill consensus conference of 2012, this patient’s AAV is most consistent with the MPA subtype due to the absence of granulomatous inflammation or eosinophils [19]. In addition, the draft classification criteria of AAV was put forward by ACR/EULAR in 2017, which is applicable to patients with small or medium vasculitis. Although it applies “subtraction method” to rule out other small vasculitis and remains to be validated, it is useful and instructive in clinical practice because it entails multiple indicators such as clinical manifestations as well as imaging, pathological, and serological features. Our case met the 2017 ACR/EULAR classification criteria of MPA. The immune deposits in the biopsy of our patient implied lupus nephritis diagnosis but did not exclude the possibility of MPA, which is classically considered to be pauci-immune, with focus of necrosis. SLE and AAV are separate diseases with different pathophysiologies and an overlap syndrome has only been reported few times in the literature, especially in the pediatric population. We present a unique clinical case of a 14-year-old girl, without a previous personal or family history of systemic autoimmune disease, with serological and biopsy findings of both diseases occurring simultaneously.

ANCA, typically p-ANCA, can be detected in patients with lupus nephritis, ranging from 3.69 to 16.9%. This finding is rarely of clinical significance since most patients do not get concomitant vasculitis. This confounding effect can be in part due to a cross reaction with ANA. Previous studies have demonstrated that classes IV and III were predominant in ANCA-positive LN. Furthermore, MPO-ANCA-positive LN patients were more likely to have lower titers of ANA, higher serum concentrations of complement, and worse baseline kidney function, all of which were consistent in our case [21–25]. Distinguishing AAV from SLE vasculitis may be difficult, as SLE vasculitis can manifest in small vessels and occurs in up to one-third of patients with underlying SLE [26]. Therefore, renal histopathological findings are paramount in making the final diagnosis.

With only few cases of overlap syndrome of SLE and AAV in previous literature, this case report provides further insight into this unique presentation with pulmonary-renal syndrome. Although AAV is rare in childhood, renal involvement is common and occurs in 60–90% of reported pediatric cases. The most severe form of renal involvement in AAV is the rapidly progressive ANCA-associated necrotizing crescentic GN. Among children, 30–40% will progress to CKD and about 10% to kidney failure by adulthood [27–29]. The prognostic factors of SLE and AAV are not invariably uniform. In 2010, an international working group of renal pathologists developed a new histopathologic classification system for ANCA-associated glomerulonephritis (focal, crescentic, mixed, and sclerotic) [30]. Noone et al. [31] has analyzed its association with renal outcomes in the largest cohort of pediatric patients (40 patients) so far. There is a significantly poor renal survival in the crescentic/mixed group and sclerotic category, with an adjusted hazard ratio of 3.14 and 23.6, respectively, compared with the focal category. Negative prognostic factors for lupus nephritis was associated with an initial Class IV histology (relative risk, 1.78), hypertension at presentation (relative risk, 1.67), and low C3 complement level in conjunction with a high creatinine level (relative risk, 1.52) [32].

A French nationwide survey was conducted to identify cases of SLE/AAV overlap syndrome and only eight cases was identified, among which only two cases occur concomitantly. A severe clinical presentation (rapidly progressive glomerulonephritis and frequent pulmonary involvement) is an almost universal feature in SLE/AAV overlap syndrome, as exhibited in our patient. In addition, a majority have both ANA and anti-MPO antibodies [33]. Previous reports of AAV/SLE overlap syndrome were depicted in Table 2 [34–42].

Considering the lack of evidence in children and the high level of evidence in adult studies for AAV, the EULAR recommendations on adult-onset AAV can be applied in pediatric AAV patients. An approach for the treatment of crescentic glomerulonephritis/rapidly progressive glomerulonephritis was described in 2018 European consensus-based recommendations for the diagnosis and treatment of rare pediatric vasculitides. The treatment is divided into two phases: a period of intense treatment (induction therapy), followed by a period of less intense maintenance therapy. Induction regimens typically include high dose corticosteroids, intravenous cyclophosphamide, and therapeutic plasmapheresis. The recommended first-line maintenance options are azathioprine, methotrexate, and mycophenolate mofetil [5]. Early diagnosis and prompt commencement of therapy is of vital important to maximize chances of renal recovery.

Our patient was initially treated with pulse doses of intravenous steroids, cyclophosphamide, and plasmapheresis. The patient is following up with the nephrology and rheumatology department and has received the additional planned cyclophosphamide infusions while taking oral hydroxychloroquine and prednisone. She has completed the sixth round of cyclophosphamide infusions and was on maintenance therapy with MMF up to now. The serum creatinine level and 24-h proteinuria declined continuously, indicating a dramatic improved kidney function as well as a favorable renal response. Besides, dramatic improvement was observed in pulmonary imaging and the hemoglobulin level has restored and remained stable in the normal range. Thus the prednisone dose was tapered gradually, with stable blood pressure. We will continue to have a follow up with the patient. Further investigations are needed to elucidate the possible shared immune pathogenesis involved in co-existence of SLE and AAV in these patients to seek for new therapeutic options.

To conclude, for patients who present with acute pulmonary-renal syndrome with atypical lupus serology (relatively lower titers of ANA, serum complement of threshold value), prominently elevated proportion of crescents in renal biopsy, along with ANCA positivity, clinicians should be aware of concurrent AAV. Proper vigilance and timely diagnosis of AAV in a lupus patient will lead to early initiation of proper disease-oriented treatment.
